# Nitric-Acid for Itching of the Nose

**Published:** 1897-01

**Authors:** J. W. Thomson

**Affiliations:** 159 West 48th St., New York


### NITRIC-ACID FOR ITCHING OF NOSE.

159 West 48th St., New York,

December 8th, 1896.

Editor of The Homœopathic Physician:

The November number of your journal is received.

You have overlooked one or two items in your editorial on Nitric-acid. Cina has more itching of nose, and stronger than any other remedy. In Nitric-acid the itching does not, I think, cause nose-bleed. In Cina the child's nose itches so that it rubs until it causes bleeding (Hepar, Croton-tig., Asaf.). Under Croton-tiglium, when there is itching it hurts to scratch. Nitric-acid has sensation as if sharp splinters were run in at the slightest touch; even of the sheet. Hydrast-can. has sensation of dropping at posterior nares, very strongly. See *Guiding Symptoms*, Vol. VI, page 57, tenth and eleventh lines from the top; also Allen's *Encyclopedia*, Vol. IV, page 618, first line, and page 619, fourth line. Nitric-acid should also be thought of in Syphilides facei. The patient has fair skin and dark hair.

J. W. Thomson.

